# Notes on Preparations for the Sick

**Published:** 1901-06

**Authors:** 


### Notes on Preparations for the Sick.

**Carnos.**—CARNOS, LIMITED, Great Grimsby.—Carnos and Carnos Invalid Extract are fluid preparations of meat, of high sp. gravity, which when diluted leave no sediment, and may be prepared hot or cold, making a valuable and palatable drink either for winter or summer. Carnos is put up in pocket flasks with screw stopper and metal cup, in a form suitable for cyclists and tourists. The clear solution remains fairly clear on boiling, but gives a copious preciptate with picric acid. Its analysis shows that it contains a high percentage of albuminoid and carbo-hydrate nourishment. It has been partially peptonised in the course of manufacture, and is therefore a soluble and easily assimilated preparation which requires no further digestion.

**Tabloids.**—BURROUGHS, WELLCOME & CO., London.—The supply of tabloids never fails, and is capable of indefinite variation. One of the most useful is the latest we have received:—

**Codeine and Nux Vomica.**—One grain of the former with a quarter grain of the latter extract. The combination is one which must at times be of use to the diabetic, and in cases of phthisis a dose at bedtime is of great service.

**Iron Citrate Compound.**—This tabloid contains three grains of citrate of iron and ammonia, with one grain of sulphate of quinine and one-sixtieth of arsenious acid. It is a good combination which must have a wide range of utility.

**Ergotin.** (Extract of Ergot, B.P.) gr. j.—This tabloid is, like the others, sugar-coated, and is made from a reliable preparation. Wherever Ergot is likely to be needed, the tabloid will be a convenient form for its presentation.

**Beta-Naphthol Compound.**—Its formula is as follows:—

| | |
|---|---|
| Beta-Naphthol - - - - | gr. j. |
| Wood Charcoal - - - - | gr. iv. |
| Oil of Peppermint - - - | ♏½. |

The combination should be of use in cases of gastric fermentation, and wherever gastric and intestinal antiseptics are likely to be useful.

**Benzoic Acid Compound.**—This tabloid is a compound of benzoic acid, codeine, menthol, ipecacuanha, cocaine, and red gum, which should give relief in cases of relaxed and irritable sore-throats.

---

**Ammonol.**—AMMONOL CHEMICAL CO., New York.—Amongst the numerous coal-tar derivatives in use a product of the amido-benzene series, called Ammonol, seems to deserve a more extended use as an antipyretic and analgesic. As it contains ammonia it is not likely to give rise to cardiac depression. It is put up in tablet form in many combinations, and has a considerable range of utility, including influenza and dysmenorrhœa.

---

**Phenalgin.**—ETNA CHEMICAL CO., New York.—This drug—ammonio-phenylacetamide—in doses of 2½ to 10 grains, easily taken in five grain capsules or in 2½ grain tablets, and repeated every half-hour, is especially recommended for cases of painful menstruation. For headache and other neuroses it is often of much service.

---

**Vasogen.**—E. J. REID, 11 Dunedin House, Basinghall Street, London, E.C.—A series of these preparations has been forwarded. Since 1893 it has been coming into use as a solvent for various drugs, which when applied to the skin becomes readily absorbed and may be found in the urine. The vasogen ointment base will take up more than 1,000 per cent. of water, and most drugs can be readily incorporated with it by trituration in a mortar without heat or any other oleaginous mixture. Compounds with creasote 20%, iodine 10%, iodoform 3%, sulphur 30%, and mercury 50%, appear to be especially useful. These compounds are manufactured by the Vasogenfabrik Pearson & Co., of Hamburg.

